# Smart glasses-assisted telementoring in inguinal hernia repair: a new frontier in surgical training?

**DOI:** 10.1007/s00464-026-13255-w

**Published:** 2026-08-24

**Authors:** Cristina Carruezzo, Marta Goglia, Chiara D’Alterio, Roberto Santoro, Angelo Goglia, Antonio Bovino, Manuela Brighi, Domenico Giannotti

**Affiliations:** 1 Department of Surgery Pietro Valdoni, Sapienza University of Roma, Rome, Italy; 2 Department of General Oncological Surgery, Santa Rosa Hospital, Viterbo, Italy; 3 Department of Medical Surgical Sciences and Translational Medicine, Faculty of Medicine and Psychology, Sapienza University of Rome, Via Di Grottarossa 1035, 00189 Rome, Italy; 4 IRCSS-Regina Elena National Cancer Institute, Rome, Italy

**Keywords:** Telementoring, Smart glasses, Surgical training, Inguinal hernia, Wearable technology, Surgical education

## Abstract

**Background:**

Recent challenges in surgical training, including the COVID-19 pandemic, staff shortages, and service centralization, have accelerated the adoption of telemedicine technologies. Among these, smart glasses-assisted telementoring enables real-time, bidirectional interaction between mentor and mentee, potentially improving surgical performance and learning outcomes. This study aimed to evaluate the efficacy and educational impact of smart glasses-assisted telementoring in open inguinal hernia repair.

**Methods:**

This prospective, randomized pilot study was conducted at the Department of General Oncological Surgery, Santa Rosa Hospital, Viterbo (Italy) from January 2024 to June 2024. Forty-five elective open inguinal hernia repairs were performed by three general surgery residents at a comparable level of their learning curve. Procedures were randomly assigned to one of three groups composed of passive assistance only, on-site mentoring and telementoring (Vuzix M400) group. Surgical and postoperative data were collected and stored for analysis.

**Results:**

Procedural accuracy was significantly higher in the mentoring settings, particularly for local anesthesia administration (Group 1: 26.7%, Group 2: 80%, and Group 3: 93.3%; *p* = 0.001) and spermatic cord isolation (Group 1: 60%, Group 2: 86.7%, and Group 3: 100%; *p* = 0.014). Mean operative times were 45.33 ± 12.88, 44.00 ± 11.21, and 60.66 ± 14.37 min in groups 1–2–3, respectively (*p* = 0.003). No statistically significant differences were detected in postoperative pain (VAS at 3 h, 24 h, and 7 days), hematoma formation, or readmissions; however, the study was not powered to assess differences in postoperative complications. Among the fifteen telementored procedures, mentees reported that telementoring influenced surgical decision-making in 93.3% of cases and consistently improved both perceived safety and self-confidence (100%), without any reported increase in anxiety or discomfort.

**Conclusions:**

Smart glasses-assisted telementoring demonstrated encouraging educational potential as a feasible approach for surgical training. These preliminary findings require confirmation in larger adequately powered studies.

**Supplementary Information:**

The online version contains supplementary material available at https://doi.org/10.1007/s00464-026-13255-w.

In recent years, healthcare systems have faced a series of challenges resulting from the Covid-19 pandemic, the shortage of medical personnel, and the centralization of services. Consequently, there has been a growing interest in the development and dissemination of new technologies applicable to the field of telemedicine. In this context, telementoring may represent an effective tool for the training of young surgeons. According to the Society of American Gastrointestinal and Endoscopic Surgeons (SAGES), *telementoring* is defined as “a relationship, facilitated by telecommunication technology, in which an expert (mentor) provides guidance to a less experienced learner (mentee) from a remote location” [1]. This innovative tutoring model relies on devices that enable visual and audio feedback and allow two-way remote interaction between mentor and mentee during surgical procedures. Smart glasses are head-mounted wearable devices equipped with a high-definition camera, microphone, and audio system. They allow hands-free live streaming of the surgical field, providing the mentor with a real-time view of the mentee’s perspective and enabling direct verbal communication through Wi-Fi connection to a remote computer or smartphone. Traditionally, surgical training during residency involves the gradual execution of procedures of increasing complexity as the first operator, under the direct supervision of an expert surgeon who provides guidance and assistance at the operating table. To evaluate the innovative approach of surgical training through telementoring, we chose open inguinal hernia repair which is one of the most common and standardized procedures performed by surgical residents. This operation requires both theoretical and technical skills that are essential for surgical training. Moreover, the open inguinal hernia repair has been widely studied in the literature as a model for assessing the surgical learning curve [2–5]. According to Nazari et al., surgical procedures can be divided into specific steps to evaluate the correctness of each stage and assess overall proficiency [6]. Continuous technological development in high-definition video acquisition and transmission systems applied to the operating room has opened new horizons in surgical education, including the possibility of remote mentoring.

Although some case series have described the feasibility of telementoring, the literature still lacks systematic studies evaluating its effectiveness in improving the learning curve of general surgery residents. The aim of this pilot study was to explore the feasibility and educational potential of smart glasses-assisted telementoring in open inguinal hernia repair, using wearable devices equipped with an integrated camera, microphone, and earphones that allow real-time, bidirectional audio-visual interaction between mentor and mentee.

## Methods

The present study was conducted at the Unit of General Oncological and Emergency Surgery, Santa Rosa Hospital, Viterbo, Italy. It was designed as a prospective, randomized pilot study. Data were collected from January 2024 to June 2024. The study was approved by the local Ethics Committee (Protocol No. 133/2024) and conducted in accordance with the Declaration of Helsinki and Good Clinical Practice guidelines. All participants, residents and patients, were enrolled voluntarily and provided written informed consent prior to participation. Written informed consent for publication of intraoperative images was obtained from all patients.

Primary outcome was intended as the procedural accuracy assessed using the study-specific OCHRA-based 12-step checklist. Secondary outcomes were measured as operative time, postoperative pain (VAS), postoperative complications (hematoma and readmission), and residents’ subjective perceptions assessed through the post-procedure questionnaire.

### Participants

Three general surgery residents (postgraduate year III–IV) participated as first operator, performing a total of 45 open inguinal hernia repairs. At the beginning of the study, each resident had performed fewer than 20 open inguinal hernia repairs as first operator, at least five of which had been completed in the same surgical Unit under the supervision of an attending senior surgeon. According to the study protocol, all three residents were at a comparable stage of training (PGY III–IV), had comparable previous experience in open inguinal hernia repair, and each performed five procedures in each study arm. This balanced allocation was intended to minimize systematic differences related to operator experience. Each resident was assisted during the procedures by attending surgeons specialized in abdominal wall surgery, all of whom had performed over 200 open inguinal hernia repairs with mesh placement as first operator.

### Study design

The surgical procedures were randomized into three different scenarios:

1. No Mentoring (Group 1)—The resident was assisted by a *passive* attending surgeon at the operating table, who provided no verbal guidance and stepped in only in case of errors, risk of injury, or failure to identify key anatomical structures.
2. On-site Mentoring (Group 2)—The resident was assisted by an *active* attending surgeon, who offered verbal suggestions and practical demonstrations during various steps of the procedure.
3. Telementoring (Group 3)—The resident, wearing smart glasses, was assisted at the operating table by a *passive* attending surgeon (as in Group 1) but received real-time verbal guidance from a remote attending surgeon (the telementor) through the audio system.

In accordance with the principles of telementoring as defined by SAGES Project 6, the telementor had previously supervised the resident in at least five open inguinal hernia repairs before the start of the study [1].

### Patients

A total of 60 adult patients with a diagnosis of symptomatic inguinal hernia were enrolled, but only 45 respected inclusion criteria and were randomly assigned to the three groups (15 patients per group). Details are reported in the Consort diagram flow (Supplementary Material) [7].

Inclusion criteria were age ≥ 18 years (male or female); diagnosis of symptomatic inguinal hernia suitable for elective open repair with mesh placement; provision of written informed consent by the patient or legal guardian. Exclusion criteria comprehended an ASA score ≥ III; a diagnosis of large inguinoscrotal (permagna) or recurrent inguinal hernia.

### Device and communication setup

The device used for remote communication between the resident and the telementor was the Vuzix M400 Smart Glasses. This monocular device features a 12.8-megapixel camera, built-in microphone, and audio system, powered by a Qualcomm Snapdragon XR1 CPU. It was connected via Wi-Fi to the mentor’s laptop, enabling real-time, high-definition image transmission and bidirectional audio interaction. The resident wore the glasses throughout the procedure, providing the mentor with a direct view of the surgical field from the operator’s perspective. The Vuzix M400 smart glasses provided real-time first-person video streaming and bidirectional audio communication between the operating resident and the remote mentor. Telestration and video recording were not available. For medicolegal and patient privacy reasons, procedures were transmitted exclusively in real time and were not recorded. The smart glasses did not modify the resident’s operative view but only transmitted the surgeon’s visual perspective to the remote mentor.

### Surgical procedure

**Fig. 1:**
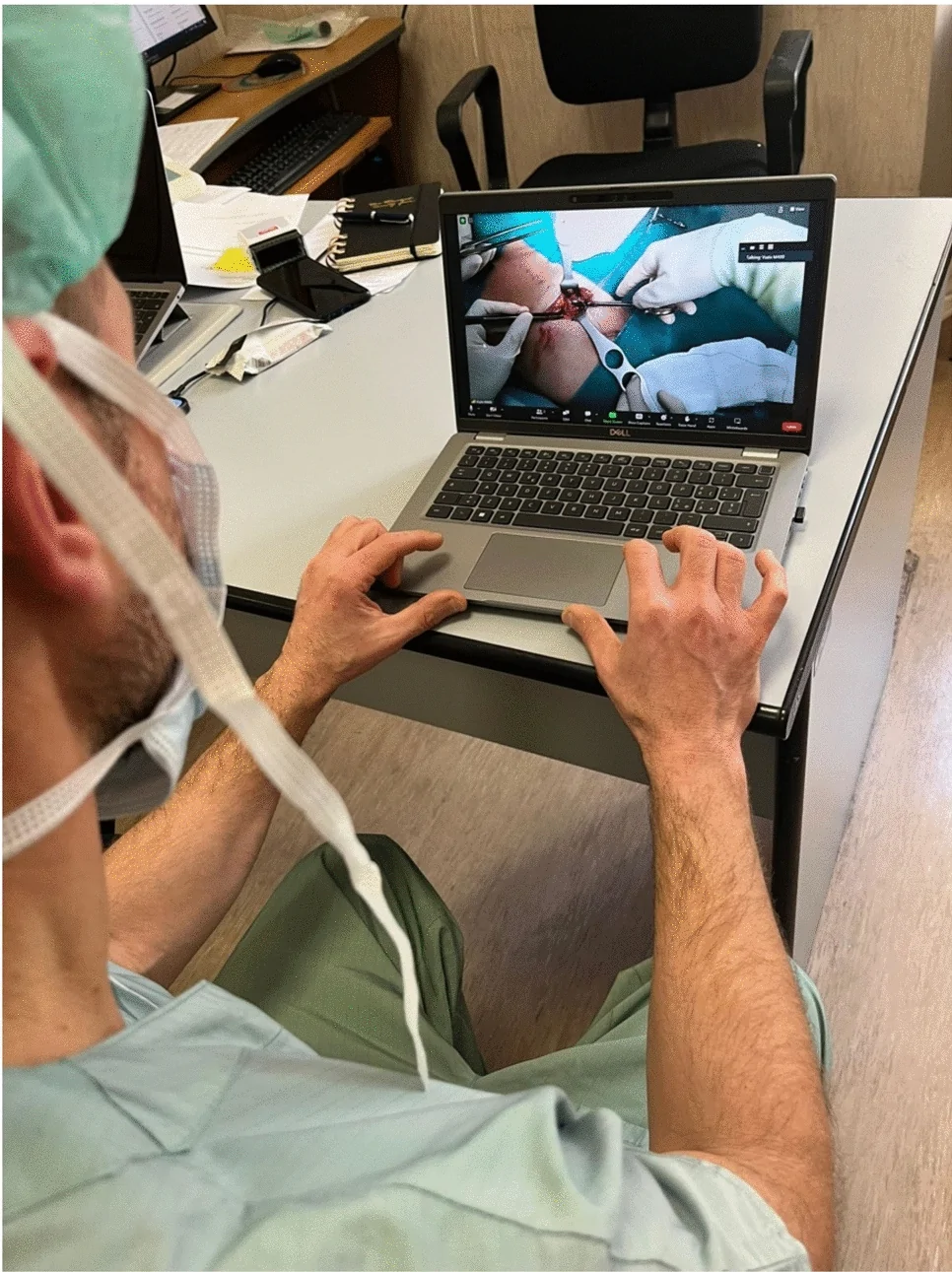
Remote mentor workstation. Remote mentor workstation during smart glasses-assisted telementoring. The telementor remotely observes the surgical field in real time through high-definition video streaming from the resident’s point of view

**Fig. 2:**
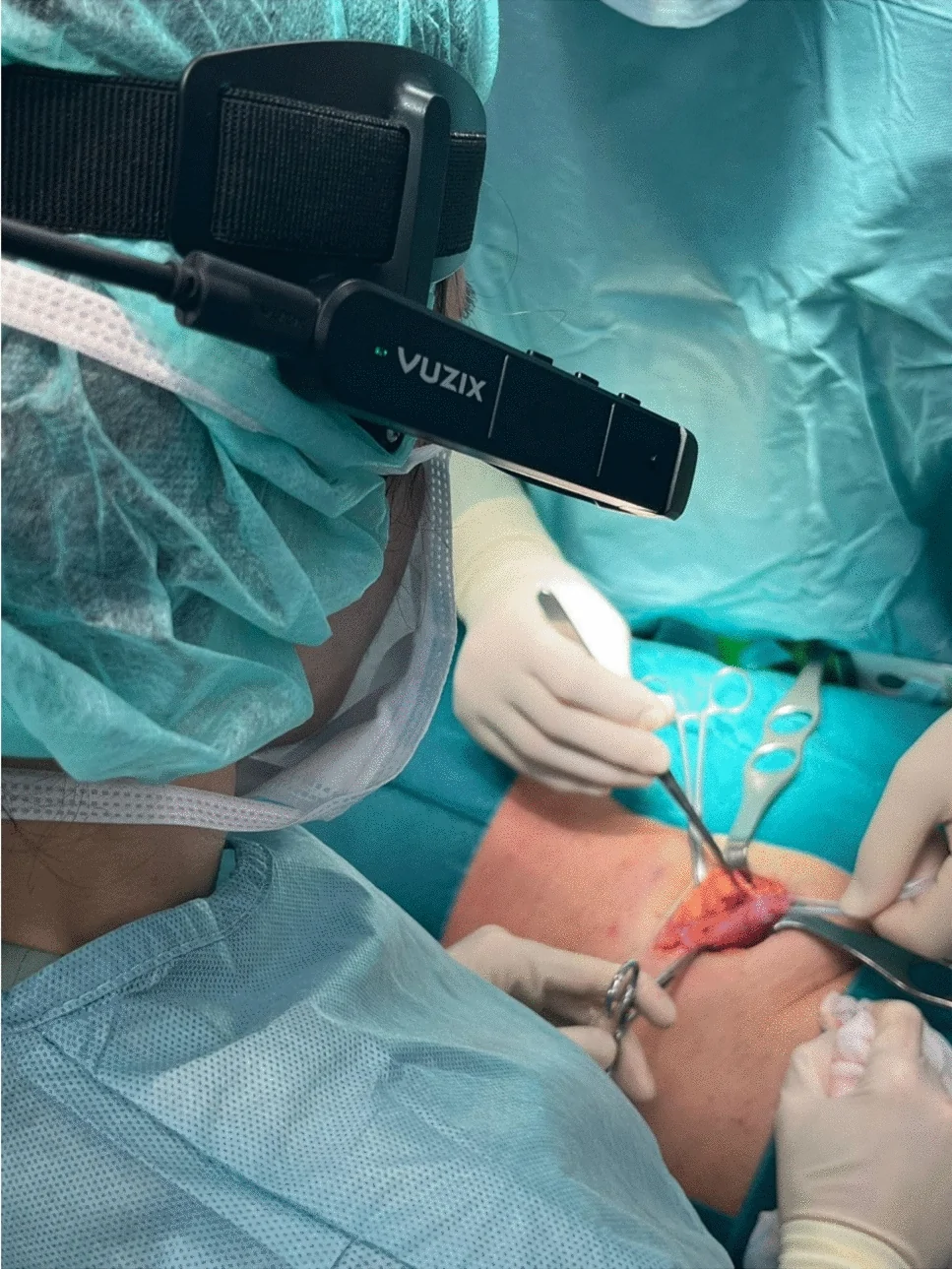
Intraoperative use of Vuzix M400 smart glasses. Intraoperative use of the Vuzix M400 smart glasses during open inguinal hernia repair. The wearable device enables hands-free audio-visual communication between the resident and the remote mentor

**Fig. 3:**
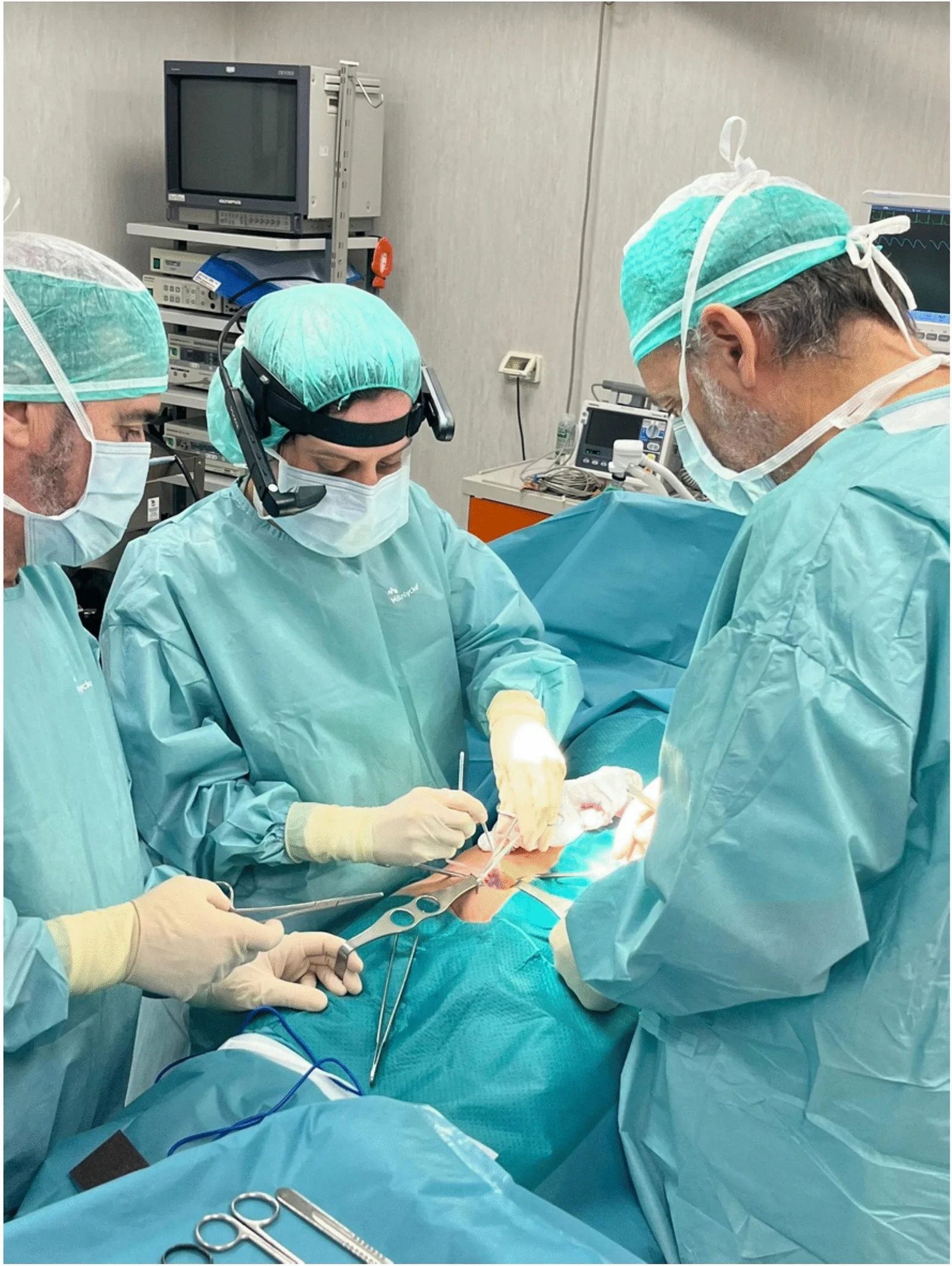
Operating room setup. Operating room setup during telementored open inguinal hernia repair. The resident wears smart glasses while performing the procedure under remote supervision and real-time guidance from the telementor

**Fig. 4:**
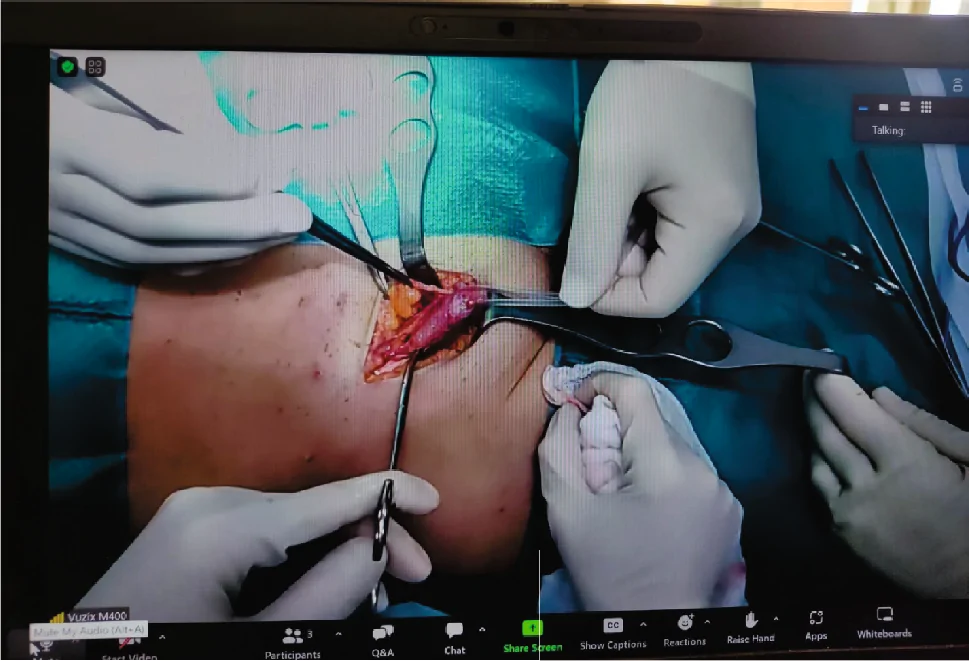
Real time surgical field visualization from the smart glasses to the remote mentor. The system allows continuous bidirectional interaction and intraoperative guidance during key procedural steps

All surgical procedures were performed under local anesthesia in a day-hospital setting, using ropivacaine and lidocaine. Skin preparation was achieved with chlorhexidine, and all patients received antibiotic prophylaxis one hour before surgery, in accordance with local institutional protocols. Mesh repair was performed using a standardized technique with lightweight polypropylene mesh. A step-by-step checklist for open inguinal hernia repair was developed based on the framework proposed by Nazari et al. [8]. The operation was divided into twelve distinct steps (Supplementary Material). The effectiveness of telementoring was assessed by comparing the three groups in terms of number and type of procedural errors per surgical step; mean operative time; postoperative pain and complications. In the Telementoring Group, additional subjective parameters were collected through a post-procedure questionnaire completed by the resident, evaluating perceived safety, self-confidence, and the perceived usefulness of remote tutoring [6]. Additional graphical details of the operative technique are presented in Figs. 1, 2, 3, and 4.

### Procedural assessment

Procedural performance was assessed using a study-specific 12-step checklist derived from the Observational Clinical Human Reliability Assessment (OCHRA) framework described by Nazari et al. The checklist incorporated the key operative phases of open inguinal hernia repair and was specifically adapted for the purposes of this study. The same predefined technical assessment criteria were applied uniformly across all three study groups. To ensure consistency of evaluation, all procedures were assessed by the same senior abdominal wall surgeon throughout the study. Depending on the study arm, the assessor was either physically present in the operating room (Groups 1 and 2) or served as the remote mentor during telementored procedures (Group 3). In Groups 1 and 3, the passive attending surgeon was instructed to intervene only when patient safety was considered at risk or when the resident was unable to safely proceed with the procedure. The checklist was completed immediately after each operation based on direct intraoperative observation. Each surgical step was classified as either correctly performed or associated with a procedural error according to the predefined technical criteria of the checklist. For the local anesthesia step, correct performance required appropriate infiltration of the planned surgical field according to the standardized operative technique, without the need for corrective guidance or additional intervention by the supervising surgeon. The same predefined criteria were applied irrespective of the study group. Therefore, the occurrence of verbal guidance or physical intervention was not considered a procedural error per se, but rather reflected the failure to achieve the predefined technical objective of that specific operative step according to the OCHRA-based assessment criteria. A procedural error was recorded when the predefined technical objective of a specific operative step was not achieved and verbal guidance or direct intervention by the supervising surgeon was required to ensure safe completion of that phase. Because mentoring itself constituted the intervention under investigation, independent blinded assessment was not feasible. Nevertheless, the use of a single assessor, a standardized assessment tool, and identical evaluation criteria across all study groups was intended to ensure consistency of procedural assessment and minimize interobserver variability.

#### Resident questionnaire

Following each telementored procedure, residents anonymously completed a study-specific questionnaire designed to explore their subjective perceptions of the telementoring experience, including perceived safety, self-confidence, communication, anxiety, comfort, and the overall usefulness of the device. The questionnaire was developed based on previous literature on telementoring and surgical education and was intended as an exploratory assessment rather than a validated educational instrument [6]. Questionnaires were completed independently after the procedure, without the mentor being present or having access to the responses.

### Data collection

#### Patient characteristics

Preoperative data included patient demographics, comorbidities, and relevant medical history, particularly the use of antiplatelet or anticoagulant therapy (Table 1).

**Table 1:** Baseline characteristics of patients in the three study groups

Variable	No mentoring	On-site mentoring	Telementoring	*p*-value
Sex (M/F)	15/0	15/0	15/0	1.000
Age (years, mean ± SD)	61.2 ± 15.6	62.7 ± 10.1	61.7 ± 9.6	0.912
Hernia type—Direct	1 (6.7%)	5 (33.3%)	3 (20%)	0.351
Indirect	13 (86.7%)	9 (60%)	12 (80%)	
Mixed (direct + indirect)	1 (6.7%)	1 (6.7%)	0 (0%)	
Side (right/left)	10/5	7/8	5/10	0.185
Antiplatelet/anticoagulant therapy (yes/no)	3/12	0/15	3/12	0.177

Each group was composed of 15 patients

No statistically significant differences were found among the three groups

Given the higher prevalence of inguinal hernia among males, all enrolled patients were male, although gender was not an exclusion criterion. Patients were categorized according to the Nyhus classification as type I–II (indirect hernia), type IIIA (direct hernia), type IIIB (combined direct and indirect hernia) [9].

#### Statistical analysis

Randomization was performed by an independent biostatistician using a simple randomization scheme (completely randomized design), as the enrolled patients were homogeneous with respect to baseline characteristics. The selection was conducted using PASS 2021 software by an independent statistician and it was concealed from the investigators until patient enrollment. Group allocation was communicated only after written informed consent had been obtained and eligibility had been confirmed, thereby preventing selection bias.

Continuous variables are reported as mean ± standard deviation (SD) or as median and interquartile range (IQR), depending on data distribution. Categorical variables are expressed as frequencies or percentages. Comparisons between continuous variables were made using the Student’s *t*-test or Wilcoxon test, as appropriate. The Fisher’s exact test or Chi-square test was used for categorical variables. Differences among groups were assessed using the non-parametric Kruskal–Wallis test, with Bonferroni correction for multiple comparisons. A *p*-value < 0.05 was considered statistically significant. All patient data were collected anonymously and stored using an alphanumeric code system.

## Results

### Patient characteristics

45 adult male patients were assigned to three groups of 15 each:

- Group 1—No Mentoring,
- Group 2—On-site Mentoring,
- Group 3—Telementoring.

Baseline demographic and clinical characteristics were comparable among the three groups (Table 1). No statistically significant differences were found in terms of age, hernia type, laterality, or use of antiplatelet/anticoagulant therapy. All procedures were performed under local anesthesia.

Procedural performance was assessed according to the standardized 12-step checklist described in the Methods. Mentoring, both on-site and remote, was associated with higher procedural accuracy compared with no mentoring (Table 2).

**Table 2:** Technical accuracy by surgical step and comparison among groups

Surgical step	Correctly performed G1/G2/G3%	*p*-value	Significant pairwise differences
Local anesthesia	26.7/80/93.3	0.0007*	G1 vs G2, G1 vs G3
Cutaneous incision	73.3/80/100	0.111	n.s. after yates correction
Isolation of spermatic cord on loop	60/86.7/100	0.0142*	G1 vs G3
Nerve identification	73.3/93.3/100	0.0537	n.s. (yates > 0.05)
Other procedural steps^†^	> 85% in all groups	> 0.05	–

Results are expressed in percentage

*G1* no mentoring, *G2* on-site mentoring, *G3* telementoring, *n.s.* not significant

^†^Includes: identification of superficial epigastric vessels, aponeurosis exposure, aponeurosis incision, cremasteric dissection, hernia sac isolation, mesh placement, and respect of vas deferens and spermatic vessels

* indicates statistical significance

Local anesthesia administration was correctly performed in 26.7% of cases in Group 1, 80% in Group 2, and 93.3% in Group 3 with a statistically significant difference between no-mentoring and both mentoring groups (*p* = 0.0007). Cutaneous incision and nerve identification were correctly executed in 73.3, 80, and 100%, and 73.3, 93.3, and 100% of cases, respectively without statistically relevant differences. On the contrary, isolation of the spermatic cord on loop showed a significant improvement in the mentored groups (60, 86.7, and 100%; *p* = 0.0142), with the difference reaching significance between the no-mentoring and telementoring groups.

For all other procedural steps, such as identification and ligation of superficial epigastric vessels, aponeurosis exposure and incision, cremasteric dissection, hernia sac isolation, mesh placement, and preservation of the vas deferens and spermatic vessels, no statistically significant differences were found among groups.

The mean operative time of the three groups were 45.33, 44.00, and 60.66 min, respectively with a significant *p* value (*p* = 0.003). Pairwise analysis showed that the telementoring group had longer operative times compared with both the no-mentoring (*p* = 0.019) and on-site mentoring groups (*p* = 0.005), suggesting that remote guidance may slightly prolong the procedure.

Concerning postoperative outcomes, no significant differences were found in postoperative pain scores, hematoma, or readmission rates among groups (Table 3). Postoperative hematoma occurred in two patients in the telementoring group (13.3%; 95% CI 1.7–40.5%) and in none of the patients in the no-mentoring or on-site mentoring groups (0%; 95% CI 0–21.8% for each group). Although this difference was not statistically significant (*p* = 0.123), the wide confidence intervals reflect the limited precision associated with the small sample size.

**Table 3:** Operative time and postoperative outcomes

Outcome	No mentoring	On-site mentoring	Telementoring	*p*-value
Operative time (min, mean ± SD)	45.33 ± 12.88	44.00 ± 11.21	60.66 ± 14.37	0.003*
VAS 3 h (mean ± SD)	1.47 ± 1.73	2.47 ± 2.39	3.00 ± 2.65	0.258
VAS 24 h (mean ± SD)	3.20 ± 2.01	3.20 ± 1.66	3.27 ± 2.66	0.978
VAS 7 days (mean ± SD)	0.67 ± 0.72	0.53 ± 1.13	0.47 ± 0.83	0.526
Postoperative hematoma (%)	0 (95% CI 0–21.8)	0 (95% CI 0–21.8)	13.3 (95% CI 1.7–40.5)	0.123
Readmission (%)	0 (95% CI 0.0–21.8%)	0 (95% CI 0.0–21.8%)	0 (95% CI 0.0–21.8%)	1.000

Postoperative pain and complication rates were comparable across all groups

*VAS* visual analogue scale, *SD* standard deviation, *CI* confidence interval

* indicates statistical significance

Regarding the Mentee’s feedback a questionnaire was completed by the residents after each telementored procedure, fifteen in total (five per resident), to qualitatively evaluate the usefulness of the technology as a surgical training tool [6]. Most mentees (80%) agreed and 13.3% strongly agreed that telementoring modified surgical strategy during specific operative steps. A positive effect on safety was reported by 73.3% (agree) and 26.7% (strongly agree) of respondents, while self-confidence was enhanced in 60% (agree) and 40% (strongly agree) of cases. No increase in performance anxiety was observed, with 73.3% disagreeing and 26.7% strongly disagreeing that the system caused stress. Similarly, 80% disagreed and 20% strongly disagreed that the device produced discomfort such as visual strain or limited head motion. All residents (73.3% agree; 26.7% strongly agree) found that the system facilitated the understanding and application of mentor suggestions. Finally, all mentees considered telementoring a useful remote tutoring tool (33.3% agree; 66.7% strongly agree). Overall, feedback was highly favorable, supporting that smart-glasses-assisted telementoring improved perceived safety, confidence, and communication without negatively affecting comfort or concentration. Details of the descriptive analysis are summarized in Table 4.

**Table 4:** Mentees’ feedback on smart glasses-assisted telementoring

Item *N* (%)	Strongly disagree	Disagree	Agree	Strongly agree
Telementoring modified surgical strategy	0	1 (6.7)	12 (80)	2 (13.3)
Increased safety during critical steps	0	0	11 (73.3)	4 (26.7)
Improved self-confidence	0	0	9 (60)	6 (40)
Increased performance anxiety	4 (26.7)	11 (73.3)	0	0
Caused physical discomfort	3 (20)	12 (80)	0	0
Facilitated application of mentor’s suggestions	0	0	11 (73.3)	4 (26.7)
Useful as a remote tutoring tool	0	0	5 (33.3)	10 (66.7)

Fifteen feedback questionnaires were collected (five per resident). Overall responses were highly positive, indicating perceived benefits in safety, confidence, and communication, with minimal discomfort or anxiety

## Discussion

This prospective randomized pilot study provides preliminary evidence that smart glasses-assisted telementoring is a feasible educational strategy for open inguinal hernia repair. Compared with procedures performed without active mentoring, both on-site mentoring and telementoring were associated with higher procedural accuracy in selected operative steps. Although operative time was longer in the telementoring group, no statistically significant differences in short-term postoperative outcomes were observed. Residents also reported favorable perceptions regarding safety, confidence, and communication. Overall, these results suggest that smart glasses-assisted telementoring has promising educational potential while maintaining comparable short-term clinical outcomes in this pilot study. The use of telecommunication technology for remote surgical guidance was pioneered by the Society of American Gastrointestinal and Endoscopic Surgeons (SAGES) through the *Project 6 Initiative*, which established telementoring as a structured educational model linking mentors and mentees across distances [1].

The current literature on telementoring demonstrates growing evidence of feasibility, safety, and efficacy, with applications ranging from surgical training to remote intraoperative consultation. Systematic reviews confirm that telementoring improves surgical skills, increases trainee confidence, and overcomes geographic and economic barriers, with positive experiences reported in laparoscopic, robotic, and open procedures [10–12]. Indeed, studies have validated its feasibility and educational value in both laparoscopic and open procedures. SAGES defined telementoring not merely as a transfer of visual and verbal information, but as a relationship built on trust, clear communication, and structured feedback, in which the mentor’s responsibility extends beyond real-time guidance to the development of the mentee’s independent decision-making and technical judgment [1]. Our study reflects this philosophy by ensuring that every remote mentor had previously established an in-person educational relationship with the resident, aligning with the Project 6 recommendations that mentorship quality should precede technological implementation. Smart glasses and augmented reality (AR) devices represent a significant evolution, enabling direct visualization of the operative field from outside the operating room for mentors and without the need to divert attention to external monitors for mentees [13, 14]. Moreover, recent studies demonstrate that the use of AR head-mounted displays significantly reduces intraoperative stress (reduction in peak heart rate from 131 to 119 bpm, *p* = 0.004) and improves procedural accuracy compared to traditional systems [13, 14]. Devices such as Microsoft HoloLens, Google Glass, and RealWear Navigator® have been successfully tested in both high- and low-income settings, showing particular utility in developing countries where the shortage of expert surgeons is critical [15–17]. Applications include real-time telementoring during cholecystectomies, colorectal resections, fundoplications, and other general surgery procedures, with high completion rates and absence of intraoperative complications [12, 18]. However, some technical limitations persist, including limited battery life, image stabilization issues, internet connection latency in remote areas, and optical resolution not always optimal for visualizing fine anatomical details [16, 19, 20]. Despite these challenges, cost–benefit analysis demonstrates that telementoring with smart glasses is more efficient than traditional in-person mentoring, particularly over extended periods [17]. Furthermore, Schlachta et al. reported that telementoring using high-definition video transmission was technically feasible and safe, with no increase in operative risk [1]. Their study emphasized the need for standardized performance metrics and structured feedback mechanisms to objectively assess its educational benefit. Our results expand upon their findings by integrating these principles with a quantitative step-by-step framework and an Observational Clinical Human Reliability Assessment (OCHRA) checklist to evaluate technical accuracy, thereby linking telementoring to measurable learning outcomes through objective performance metrics. Similarly, Nazari et al. validated this approach in open inguinal hernia repair, showing that dividing procedures into standardized steps improves both teaching reproducibility and assessment reliability [6]. Our study extends previous work by combining structured procedural assessment with prospective randomized evaluation of smart glasses-assisted telementoring. Indeed, in the broader context of surgical innovation, Lareyre et al. highlighted the advantages of smart glasses and head-mounted displays (HMDs) for intraoperative communication and real-time teaching, while acknowledging current limitations such as video resolution and device ergonomics [21]. This study suggests that such wearable technology can be seamlessly incorporated into general surgery without compromising workflow or safety. Other recent studies have emphasized that effective telementoring depends not only on technology, but also on the interpersonal dynamics that allow real-time guidance to become genuine teaching. When trust and communication are maintained, the virtual distance between mentor and mentee ceases to be a limitation and becomes instead a catalyst for reflection, self-assessment, and independent learning [22–24]. Indeed, in accordance with our results, telementoring maintains procedural quality and enhances the educational experience, even in a standardized open surgical model. Finally, advances in immersive and AR systems have demonstrated similar benefits in cognitive engagement and technical performance during surgical training [10, 25].These technologies, together with smart glasses, are converging toward a unified vision of connected, interactive, and data-driven surgical education.

### Limitations

This study has several limitations that should be considered when interpreting the findings. First, it was designed as a single-center pilot randomized study with a relatively small sample size, limiting statistical power, external validity, and the ability to detect differences in relatively infrequent postoperative complications. Furthermore, the study was not designed or powered to demonstrate educational equivalence or non-inferiority between telementoring and conventional on-site mentoring. Although the participating residents were selected at comparable stages of training, had similar previous experience in open inguinal hernia repair, and each performed an equal number of procedures in every study arm according to the study protocol, repeated observations within the same operator were not fully independent, and residual operator-related variability cannot be excluded. Procedural assessment was performed by the same senior surgeon throughout the study, and independent blinded assessment was not feasible because mentoring itself constituted the intervention under investigation. Although the use of a single assessor and standardized assessment criteria ensured consistency across groups, the possibility of detection bias should be acknowledged. In addition, the resident questionnaire was specifically developed for this study and explored subjective perceptions rather than objective educational outcomes. Despite anonymous and independent administration, the limited number of participating residents may have introduced response bias. Longer operative times observed in the telementoring group may reflect the educational interaction, adaptation to the device, and familiarization with the communication workflow, although a formal learning curve analysis was not performed. Moreover, only one standardized open surgical procedure was evaluated; therefore, these findings may not be directly generalizable to more complex procedures or minimally invasive surgery. Finally, although no device-related technical issues occurred during the study, wearable telementoring systems may be influenced by factors such as wireless connectivity, field-of-view limitations, battery life, and video quality, which should be considered when implementing this technology in different surgical environments. Future multicenter randomized studies including larger cohorts, independent outcome assessment, longer follow-up, and objective measures of skill acquisition and retention are warranted to confirm these preliminary findings.

### Future perspectives

Telementoring represents a transformative model for surgical education, enabling equitable access to expert guidance regardless of geographical constraints. Its feasibility and safety are well established in literature; the present study suggests its educational effectiveness in a real-world, structured setting [1]. Following the *Project 6 Summit* recommendations, future implementations should continue to prioritize the human dimension of mentorship—trust, mutual respect, and feedback—while leveraging technological progress to enhance interaction quality. Beyond residency training, telementoring platforms can also serve as valuable tools for peer-to-peer consultation among experienced surgeons. Previous studies have suggested that remote surgical guidance may be used not only for didactic purposes, but also for real-time decision-making and technical support during complex or emergency procedures [1, 23, 24]. In this context, wearable systems such as smart glasses may facilitate rapid intraoperative communication between specialists, allowing expert input when facing unexpected findings, rare anatomies, or critical intraoperative events. This approach could be particularly valuable in low-resource or peripheral hospitals, where immediate on-site expert support is not always available. With ongoing advances in connectivity, augmented reality, and artificial intelligence, wearable telementoring systems may soon provide real-time analytics, performance tracking, and context-aware guidance. Such tools could be integrated into residency curricula, continuing medical education, and credentialing systems, fostering a culture of global mentorship and surgical collaboration. Ultimately, smart glasses-assisted telementoring may redefine how surgical competence and decision-making are shared, taught, and supported, bridging the gap between distance and expertise. However, the literature highlights the need for more rigorous randomized clinical trials and standardization of technologies and educational curricula to consolidate the integration of these technologies into daily surgical practice [20, 26, 27].

## Conclusions

This pilot randomized study suggests that smart glasses-assisted telementoring is a feasible and acceptable approach to support surgical training. Although residents reported favorable perceptions regarding safety, confidence, and usability, and no statistically significant differences in short-term postoperative outcomes were observed, these findings should be considered preliminary. Larger multicenter randomized studies with independent outcome assessment and longer follow-up are required to confirm the educational effectiveness of telementoring and determine its role relative to conventional on-site mentoring.

## Supplementary Information

Below is the link to the electronic supplementary material.

Supplementary file1 (DOCX 27 KB)

Supplementary file2 (DOCX 16 KB)
