# Supplementary material for: Smart glasses-assisted telementoring in inguinal hernia repair: a new frontier in surgical training?
Source: Surg Endosc. 2026 Aug 24;40(9):8128–38. doi: 10.1007/s00464-026-13255-w (PMC13582260; doi:10.1007/s00464-026-13255-w)
Supplement: Supplementary file 1 [file 464_2026_13255_MOESM1_ESM.docx]

**Figure 1. CONSORT 2025 Flow Diagram**

Flow diagram of enrollment, allocation, follow-up, and analysis in the three-arm randomized pilot study

| **ENROLLMENT** | **Assessed for eligibility (n = 60)** | Excluded (n = 15) • Large/permagna inguinal hernia (n = 7) • Declined to participate (n = 3) • Recurrent inguinal hernia (n = 3) • Other reasons (n = 2) |
| --- | --- | --- |

↓

**Randomized (n = 45)**

↓

| **ALLOCATION** | | |
| --- | --- | --- |
| No Mentoring Allocated to intervention (n = 15) Received allocated intervention (n = 15) Did not receive allocated intervention (n = 0) | On-site Mentoring Allocated to intervention (n = 15) Received allocated intervention (n = 15) Did not receive allocated intervention (n = 0) | Telementoring Allocated to intervention (n = 15) Received allocated intervention (n = 15) Did not receive allocated intervention (n = 0) |

↓

| **FOLLOW-UP** | | |
| --- | --- | --- |
| No Mentoring Discontinued intervention (n = 0) Lost to follow-up for primary outcome (n = 0) | On-site Mentoring Discontinued intervention (n = 0) Lost to follow-up for primary outcome (n = 0) | Telementoring Discontinued intervention (n = 0) Lost to follow-up for primary outcome (n = 0) |

↓

| **ANALYSIS** | | |
| --- | --- | --- |
| No Mentoring Analysed for primary outcome (n = 15) Excluded from analysis (n = 0) | On-site Mentoring Analysed for primary outcome (n = 15) Excluded from analysis (n = 0) | Telementoring Analysed for primary outcome (n = 15) Excluded from analysis (n = 0) |

*CONSORT 2025 flow diagram adapted for a three-arm randomized pilot trial.*

Adapted from: Hopewell S, Chan AW, Collins GS, Hróbjartsson A, Moher D, Schulz KF, et al. CONSORT 2025 Statement: updated guideline for reporting randomised trials. BMJ. 2025;388:e081123.
