# Supplementary material for: Smart glasses-assisted telementoring in inguinal hernia repair: a new frontier in surgical training?
Source: Surg Endosc. 2026 Aug 24;40(9):8128–38. doi: 10.1007/s00464-026-13255-w (PMC13582260; doi:10.1007/s00464-026-13255-w)
Supplement: Supplementary file 2 [file 464_2026_13255_MOESM2_ESM.docx]

**Supplementary Material – Surgical Steps**

| **Surgical steps** | | **Correctly performed** | **Procedural error** |
| --- | --- | --- | --- |
| 1 | Local anesthesia administration |  |  |
| 2 | Skin incision |  |  |
| 3 | Identification and ligation of superficial epigastric vein |  |  |
| 4 | External oblique aponeurosis exposure and inguinal canal exposure |  |  |
| 5 | External oblique aponeurosis incision |  |  |
| 6 | Isolation of the spermatic cord on loop |  |  |
| 7 | Cremaster muscle dissection |  |  |
| 8 | Hernia sac identification and isolation |  |  |
| 9 | Identification and preservation of the vas deferens |  |  |
| 10 | Identification and preservation of the spermatic vessels |  |  |
| 11 | Hernia repair with mesh placement |  |  |
| 12 | Nerve identification |  |  |
